# *Bacillus cereus* in Packaging Material: Molecular and Phenotypical Diversity Revealed

**DOI:** 10.3389/fmicb.2021.698974

**Published:** 2021-07-12

**Authors:** Paul Jakob Schmid, Stephanie Maitz, Clemens Kittinger

**Affiliations:** Diagnostic and Research Institute of Hygiene, Microbiology and Environmental Medicine, Medical University of Graz, Graz, Austria

**Keywords:** *Bacillus cereus*, phylogenetic affiliation, bacterial growth, toxins, packaging material

## Abstract

The *Bacillus cereus* group has been isolated from soils, water, plants and numerous food products. These species can produce a variety of toxins including several enterotoxins [non-hemolytic enterotoxin (Nhe), hemolysin BL (Hbl), cytotoxin K, and enterotoxin FM], the emetic toxin cereulide and insecticidal Bt toxins. This is the first study evaluating the presence of *B. cereus* in packaging material. Among 75 different isolates, four phylogenetic groups were detected (II, III, IV, and VI), of which the groups III and IV were the most abundant with 46.7 and 41.3%, respectively. One isolate was affiliated to psychrotolerant group VI. Growth experiments showed a mesophilic predominance. Based on PCR analysis, *nhe* genes were detectable in 100% of the isolates, while *hbl* genes were only found in 50.7%. The cereulide encoding gene was found in four out of 75 isolates, no isolate carried a crystal toxin gene. In total, thirteen different toxin gene profiles were identified. We showed that a variety of *B. cereus* group strains can be found in packaging material. Here, this variety lies in the presence of four phylogenetic groups, thirteen toxin gene profiles, and different growth temperatures. The results suggest that packaging material does not contain significant amounts of highly virulent strains, and the low number of cereulide producing strains is in accordance with other results.

## Introduction

The *Bacillus cereus* group, also called *Bacillus cereus sensu lato* (*B. cereus* s.l.) comprises at least eight different gram-positive, aerobic and endospore-forming species, including *B. cereus sensu stricto* (s.s.), *B. thuringiensis*, *B. anthracis, B. weihenstephanensis*, and *B. cytotoxicus* (Liu et al., 2015). They are ubiquitous and have been frequently reported in soil, sediments, water, and plants in a variety of natural environments (Von Stetten et al., 1999; Stenfors Arnesen et al., 2008; Brillard et al., 2015) as well as from fresh vegetables, rice, spices, raw meat and meat products, fish and seafood, dairy products and ready-to-eat foods (Konuma et al., 1988; Granum et al., 1993; Rahmati and Labbe, 2008; Samapundo et al., 2011; Carter et al., 2018; Fiedler et al., 2019; Yu et al., 2020). It has long been known, that packaging material may provide a reservoir for microorganisms (Välsänen et al., 1991) and regarding its widespread distribution, the occurrence of *B. cereus* group species in packaging material is more than obvious and has already been published (Suihko et al., 2004; Ekman et al., 2009). *B. cereus* is a causative agent for food spoilage and food poisoning. In particular, two types of gastrointestinal syndromes are associated to *B. cereus*, the diarrheal type caused by enterotoxins and the emetic type caused by the plasmid encoded toxin cereulide (Stenfors Arnesen et al., 2008). There are numerous enterotoxins produced by species in the *B. cereus* group, of which hemolysin BL (Hbl), the non-hemolytic enterotoxin (Nhe), cytotoxin K (CytK) and enterotoxin FM (EntFM) are among the most investigated. Hbl is a three-component pore-forming complex encoded in the operon *hblDAC* leading to cytotoxic effects, necrosis and watery diarrhea (Beecher et al., 1995). Nhe is another three-component pore-forming toxin encoded in the operon *nheABC* and showed cytotoxic effects on different human cell lines (Jeßberger et al., 2014). For both, Hbl and Nhe, all three components are necessary for full toxin activity (Beecher et al., 1995; Lindbäck et al., 2010). CytK is a beta-barrel pore-forming toxin encoded by the gene *cytK* and resembles the beta-toxin of *Clostridium perfringens* (Lund et al., 2000). EntFM was identified in *B. cereus s.s.* and *B. thuringiensis* and is a putative cell wall peptidase important for virulence (Asano et al., 1997; Tran et al., 2010). The emetic toxin cereulide (*ces* gene cluster) is a small heat- and acid-stable dodecadepsipeptide, which is synthesized by a non-ribosomal peptide synthetase (Ehling-Schulz et al., 2019). Research showed that enterotoxins are not limited to *B. cereus* s.s. but have been found in strains of *B. thuringiensis* (Ngamwongsatit et al., 2008), *B. weihenstephanensis* (Baron et al., 2007), *B. mycoides*, and *B. pseudomycoides* (Prüß et al., 1999). This is of relevance as former apathogenic species such as *B. thuringiensis* are also used as biopesticides through its plasmid encoded insecticidal crystal (Cry) or Bt toxin (Schnepf et al., 1998). This study aims to provide a comprehensive overview of *B. cereus* in packaging materials. For this purpose, 188 different samples were analyzed for the presence of *B. cereus*. In total, 75 isolates were selected for further characterization. All isolates were clustered into seven phylogenetic groups (I–VII) by partial *panC* sequencing according to Guinebretière et al. (2010) followed by maximum likelihood analysis. Furthermore, growth and hemolysis phenotypes were determined. The presence of enterotoxin genes was assessed by multiplex-PCR targeting *hblDAC*, *nheABC*, *cytK*, and *entFM*, and all isolates were tested for harboring the *ces* and *cry1*-type toxin genes. To our knowledge, this is the first study evaluating the presence of *B. cereus* in different samples of packaging material in terms of phylogeny, growth temperatures and toxin genes. Additionally, we aim to compare the results with previous data of environmental and food isolates to provide a better understanding of *B. cereus* occurring in the packaging industry.

## Materials and Methods

### Samples

In this study, 188 samples of fiber-based packaging material were included. To provide a diverse view on packaging materials, the samples were manufactured in six different European packaging material facilities. All samples came from the food packaging sector reflecting primary and secondary food packaging with different applications (Table 1). The samples were taken under sterile conditions by instructed workers and wrapped in sterile aluminum foil to avoid contamination. Afterwards the samples were sent to the laboratory in sealed plastic bags.

**TABLE 1:** Packaging material samples and corresponding applications.

Packaging material manufacturer	Number of samples	Food packaging applications
1	63	Dry, moist, fatty
2	5	Dry, moist, fatty
3	4	Dry, moist, fatty and non-fatty
4	5	Dry, reduced fat contact
5	15	Dry, fatty
6	96	Secondary food packaging
	*n* = 188	

*The samples were provided by six packaging material facilities. The packaging applications are according to the manufacturer’s specifications.*

### Determination and Isolation of *B. cereus*

One gram of each sample was weighed and evenly homogenized with 99 ml of sterile buffered peptone water (Thermo Fisher Scientific, Waltham, United States) in sterile bags using a Bagmixer (Interscience, St. Nom la Bretèche, France). Afterwards, 0.5 ml of the sample suspension were transferred on Brilliance Bacillus cereus agar (Thermo Fisher Scientific) followed by incubation at 37°C for 48 h. Homogenized samples in buffered peptone water were additionally enriched at 37°C for 24°C and streaked again on Brilliance Bacillus cereus agar to detect low numbers of *B. cereus*. All samples were tested in duplicates. A total of 75 *B. cereus* group isolates with varying colony morphologies were isolated from 41 different samples and selected for further characterization. Those 41 samples were randomly selected representing four of the six sampling sites with the highest abundance of *B. cereus* (Table 2). On the selective agar, each colony with a differing morphology was picked. Clonal isolates were excluded after *panC* sequencing and toxin gene detection. The *B. cereus* group was confirmed by MALDI-TOF (VITEK^®^ MS, bioMérieux Marcy-l’Étoile, France). Isolates were stored at –80°C for further experiments using the Microbank system (Pro-Lab Diagnostics, Richmond Hill, Canada).

**TABLE 2:** *Bacillus cereus* in packaging materials from six different manufacturers.

Packaging material manufacturer	Samples with *B. cereus* detectable in the direct assessment	Samples with *B. cereus* detectable after enrichment	Number of *B. cereus* isolates for characterization
1	1/63 (1.6%)	36/63 (57.1%)	15
2	1/5 (20.0%)	4/5 (80.0%)	3
3	0/4	1/4 (25.0%)	–
4	0/5	4/5 (80.0%)	–
5	0/15	10/15 (66.7%)	5
6	94/96 (97.9%)	96/96 (100.0%)	55
	96/188 (51.1%)	152/188 (80.9%)	*n* = 75

**Bacillus cereus* was detected in a direct assessment and after enrichment on selective medium. A total of 75 isolates were collected from samples from four different origins for further characterization.*

### Characterization of Growth and Hemolysis

All isolates were tested for growth at different temperatures (20, 37, 45, and 55°C) using the Bioscreen C growth monitoring system (Oy Growth Curves Ab Ltd., Helsinki, Finland). Isolates were grown on COL-S blood agar (Thermo Fisher Scientific) and colonies were suspended in sterile TSB (Thermo Fisher Scientific) to make a bacterial suspension equal to 0.5 McFarland standard. Afterwards, 2 μl of the suspension were incubated in 200 μl TSB for 72 h at the set temperature including continuous shaking. Measurement of the optical density at 600 nm (OD_600_) was done automatically every 15 min. All isolates were tested in technical duplicates. Due to a limited range for low temperatures, growth at 5 to 7°C was tested by streaking isolates on COL-S agar plates and subsequent incubation in the refrigerator for 7 days. To assess hemolysis, isolates were streaked on COL-S and examined for beta-hemolysis after incubation at 30°C for 24 h.

### DNA Extraction

Isolates were grown in TSB at 30°C overnight and DNA was extracted using the innuPREP Bacteria DNA Kit (Analytik Jena, Jena, Germany). Successful extraction was verified by measuring the amount of extracted DNA using the Nanodrop2000 (Thermo Fisher Scientific). The DNA was stored in elution buffer at –20°C until needed.

### Partial *panC* Sequencing

Partial *panC* gene was amplified using following primer sequences for groups I to VI according to Guinebretière et al. (2010): 5′-TYGGTTTTGTYCCAACRATGG-3′ (forward degenerated primer) and 5′-CATAATCTACAGTGCCTTTCG-3′ (reverse primer). PCR was carried out in a Biometra Trio 48 thermocycler (Analytik Jena, Germany) in a final volume of 25 μl containing 1X NEB Q5 Reaction Buffer (New England Biolabs, Ltd, Ipswich, United States), 200 μM of each dNTP, 0.5 μM of each primer, 0.5 IU of NEB Q5 High Fidelity DNA Polymerase (New England Biolabs) and 1 μl DNA template. The temperature protocol included an initial denaturation at 98°C for 30 s followed by 30 cycles of 98°C for 10 s, 61°C for 30 s, 72°C for 30 s and a final extension at 72°C for 2 min. The INVISORB Spin DNA Extraction Kit (Invitek, Berlin, Germany) was used for DNA purification and products were sent to Eurofins Genomics Germany GmbH (Ebersberg, Germany) for sequencing using primer 5′-ATAATCTACAGTGCCTTTCG-3′ (Guinebretière et al., 2010). Sequence data analysis was done by uploading to an online algorithm^1^ for assignment to groups I to VII (Guinebretière et al., 2010).

### PCR Assays for Toxin Genes

Enterotoxin genes of the *B. cereus* group were tested using a multiplex PCR assay with specific primers for *hblD*, *hblA*, *hblC*, *nheA*, *nheB*, *nheC*, *cytK*, and *entFM* (Thaenthanee et al., 2005; Ngamwongsatit et al., 2008). The multiplex PCR was performed in a final volume of 25 μl containing 1X NEB Q5 Reaction Buffer (New England Biolabs), 400 μM of each dNTP, 0.2 μM (*nheABC, cytK*, and *entFM*), 0.4 μM (*hblDA*), and 4 μM (*hblC*) of primers, 0.5 IU of NEB Q5 High Fidelity DNA Polymerase (New England Biolabs Ltd, Ipswich, United States) and 1 μl DNA template. DNA templates were tested twice, undiluted and in 1:10 dilution to minimize amplification inhibition. The temperature protocol included an initial denaturation at 98°C for 2 min followed by 40 cycles of 98°C for 30 s, 58°C for 1.5 min, 72°C for 2 min and a final extension at 72°C for 5 min. *B. cereus* ATCC 14579 was used as positive control. *Ces*-gene PCR was carried out according to CSA Group Corporation (2018). PCR was performed in a final volume of 25 μl containing 1X NEB Q5 Reaction Buffer (New England Biolabs), 200 μM of each dNTP, 0.5 μM of each primer, 0.5 IU of NEB Q5 High Fidelity DNA Polymerase (New England Biolabs) and 1 μl DNA template. *B. cereus* DSM 4312 served as positive control for *ces*-PCR. The detection of *cry1*-type genes used primer sequences and PCR protocol previously described by Choo et al. (2007). *B. thuringiensis* ATCC 10792 served as positive control for *cry1*-type gene detection. Each PCR was carried out in a Biometra Trio 48 thermocycler (Analytik Jena). Amplicons were separated on 1.6% agarose gels using a 100 bp DNA molecular size standard (New England Biolabs) for enterotoxin multiplex PCR and *ces*-PCR and a 1 kb DNA molecular size standard for *cry1*-type PCR to estimate the size of the PCR products. Agarose gels were photographed using the AlphaImager^TM^ Imaging System (Biozym, Germany) and brightness and contrast were adjusted as necessary. All target genes for PCR were sent once to Eurofins Genomics Germany GmbH (Ebersberg, Germany) for sequencing to verify amplification specificity.

### Data Analysis

All statistical data analyses were done in GraphPad Prism version 7.0.0 for Windows (GraphPad Software, San Diego, United States). Normality testing of the data was performed using the D’Agostino and Pearson omnibus normality test and the Shapiro–Wilk normality test, followed by the Wilcoxon matched-pairs signed rank test, a non-parametric test for differences in paired observations and the Kruskal–Wallis test, a non-parametric test for two or more independent medians. For the phylogenetic tree, sequences were first aligned using M-Coffee on the T-coffee server (Moretti et al., 2007) and followed by bootstrapping (100 bootstrap samples), maximum-likelihood analysis and consensus tree creation with the PHYLIP package (Felsenstein, 2005). SplitsTree was used for tree visualization (Huson and Bryant, 2006).

## Results

### Determination of *B. cereus* in Packaging Material

Bacillus *cereus* was detected in 152 out of 188 packaging material samples (80.9%). In the direct assessment by plating 0.5 ml homogenized sample, however, *B. cereus* was detectable in only 96 samples (51.1%). In another 56 samples, we could detect *B. cereus* only after 24 h enrichment in buffered peptone water. The overall number of positive samples per manufacturer revealed an uneven distribution of *B. cereus* in packaging material samples (Table 2). We detected *B. cereus* in over 90% of the samples from manufacturer 6 in the direct assessment, but only at minimal rates in the samples from the manufacturers 1 to 5. The MALDI-TOF analysis confirmed all isolates selected for further characterization as members of the *B. cereus* group.

### Affiliation to Phylogenetic *panC* Groups

**FIGURE 1:**
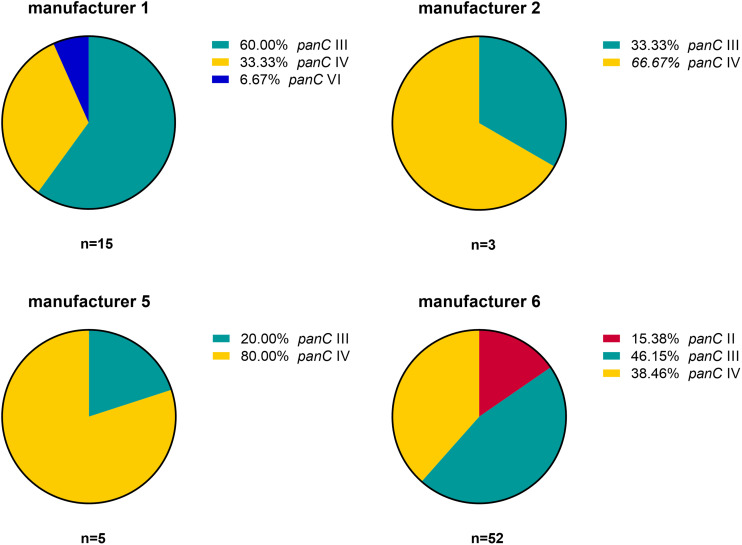
Distribution of *B. cereus* phylogenetic *panC* groups in samples from four different packaging manufacturers: II (red), III (turquoise), IV (yellow), and VI (blue).

**FIGURE 2:**
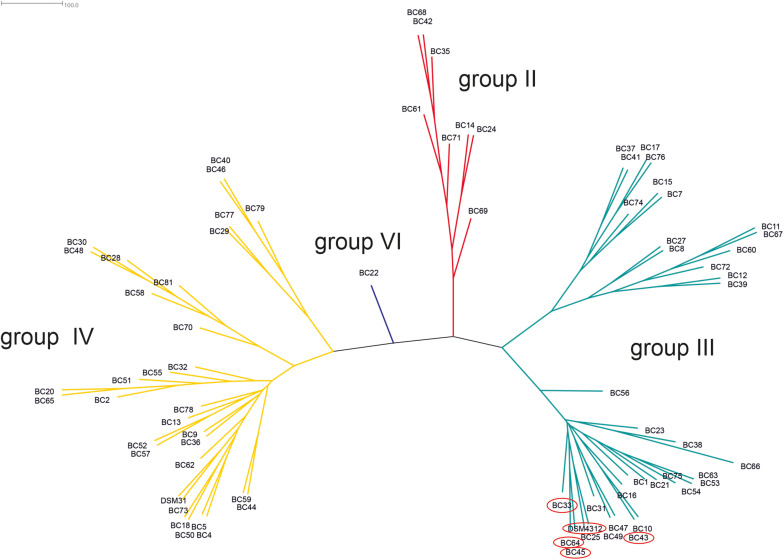
Phylogenetic tree of *B. cereus* group isolates from packaging material. Unrooted consensus tree based on maximum likelihood of partial *panC* gene sequences. Four *panC* groups were detected: II (red, *n* = 8), III (turquoise, *n* = 35), IV (yellow, *n* = 31), and VI (blue, *n* = 1). Reference strains: DSM31 (ATCC14579, group IV), DSM4312 (group III). Emetic isolates (highlighted in red): BC33, BC43, BC45, BC64, and DSM4312.

Based on the sequence analysis of the partial *panC* gene, we were able to affiliate all 75 isolates to four phylogenetic *panC* groups (II, III, IV, and VI) as defined by Guinebretière et al. (2008). Among those, mesophilic *panC* groups III and IV were the most prevalent with 35 (46.7%) and 31 isolates (41.3%), respectively (Table 3), followed by eight isolates (10.7%) affiliated to *panC* group II. A single isolate (BC22) was found to be in phylogenetic group VI, which includes mainly psychrotolerant species of the *B. cereus* group. The distribution of the phylogenetic groups in samples from different manufacturers unveiled that the *panC* groups 3 and 4 formed the majority in the samples from all manufacturers (Figure 1). The *panC* group II was only found in samples from manufacturer 6, whereas the *panC* group VI was only detected in a single sample from manufacturer 1. Additionally, a phylogenetic tree based on bootstrap analysis and maximum-likelihood shows concordant genetic clusters for each phylogenetic *panC* group determined (Figure 2). Here, *B. cereus* reference strains ATCC 14579 and DSM 4312 were included for group IV and III, respectively. Isolates possessing the emetic toxin cereulide were exclusively affiliated to group III and are phylogenetically close to reference strain DSM 4312. The phylogenetic tree revealed uniformly distributed isolates within group II, whereas group III and IV appear to be split into subgroups.

**TABLE 3:** Phylogenetic affiliation of the isolates.

Phylogenetic group	No. of isolates (%)	Corresponding species^a^	Growth characteristics^a^
II	8 (10.7%)	*B. cereus* (8) *B. thuringiensis* (0)	Mesophilic/psychrotolerant 7–40°C
III	35 (46.7%)	*B. cereus* (35) *B. thuringiensis* (0) *B. anthracis* (0)	Mesophilic 15–45°C
IV	31 (41.3%)	*B. cereus* (31) *B. thuringiensis* (0)	Mesophilic 10–45°C
VI	1 (1.3%)	*B. weihenstephanensis* (1) *B. mycoides* (0) *B. thuringiensis* (0)	Psychrotolerant 5–37°C

*^*a*^ species and growth characteristics according to Guinebretière et al. (2010). Further species classification within the phylogenetic groups was aimed using toxin gene PCR results (*cry-1* type toxin gene PCR for *B. thuringiensis*) and colony morphology (rhizoid growth for *B. mycoides*).*

### Growth and Hemolysis

**FIGURE 3:**
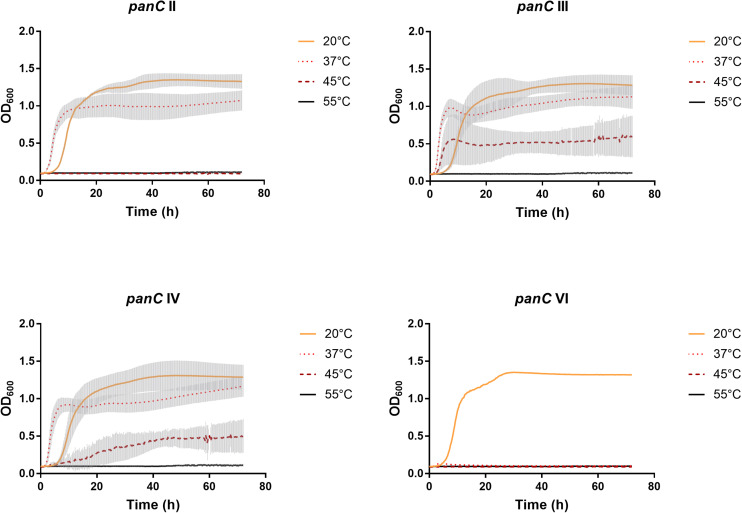
Growth curves for phylogenetic *panC* groups II, III, IV, and VI. Growth curves in TSB medium were determined at 20°C (yellow), 37°C (orange dotted), 45°C (red dotted), and 55°C (black). Phylogenetic groups: II (*n* = 8), III (*n* = 35), IV (*n* = 31), and VI (*n* = 1). The curves show means with standard deviations (gray).

**FIGURE 4:**
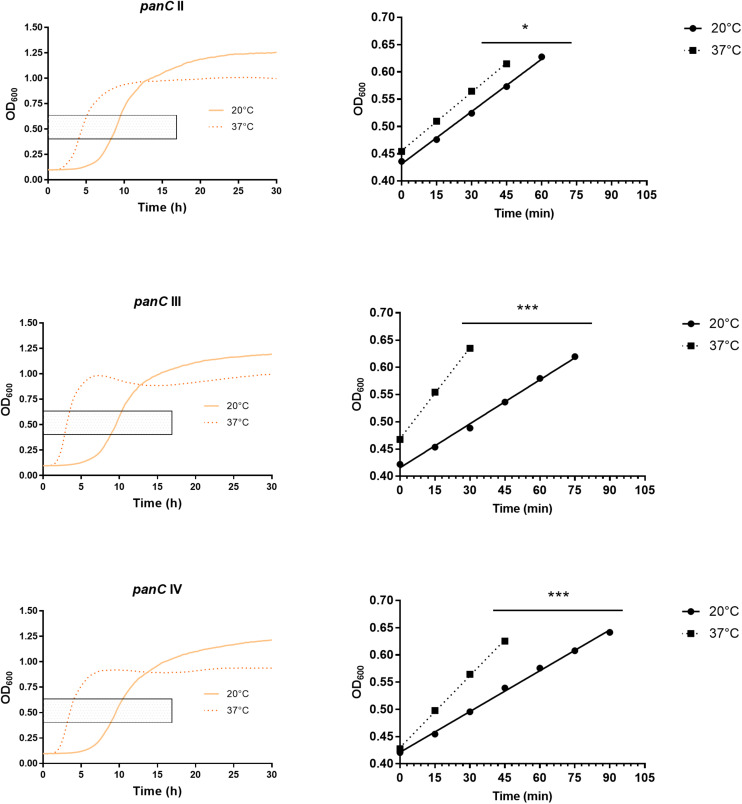
Increased growth rates during exponential phase at 37°C compared to 20°C for phylogenetic *panC* groups II, III, and IV. Left: growth curves for 20°C (yellow) and 37°C (orange dotted). Exponential phase was defined for OD600 values between 0.4 and 0.65 (gray box). Right: linear equations were modeled using OD600 values between 0.4 and 0.65 for 20°C (dots) and 37°C (boxes). Slope differences were statistically significant for each phylogenetic group: II (*p* = 0.0449), III (*p* < 0.0001), and IV (*p* < 0.0001). * indicates statistical significance with a *p*-value below 0.05. *** indicates statistical significance with a *p*-value below 0.0001.

**FIGURE 5:**
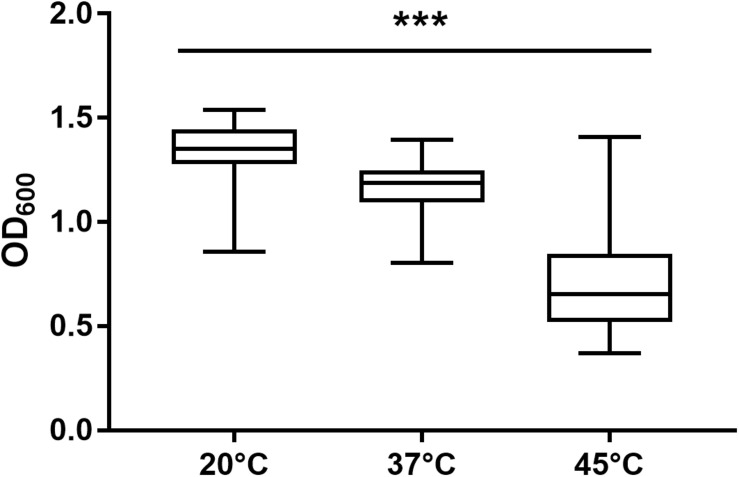
Maximum biomass yield differed between incubation temperatures of 20, 37, and 45°C. Isolates able to grow at 20°C (*n* = 75), 37°C (*n* = 74), and 45°C (*n* = 43) in TSB showed significant differences in maximum biomass yield (*p* < 0.0001). *** indicates statistical significance with a *p*-value below 0.0001.

After 72 h in the Bioscreen C, growth was detectable for all 75 isolates at 20°C (Figure 3) while isolates of mesophilic *panC* groups II, III and IV did grow at both temperatures, 20 and 37°C, the isolate affiliated to psychrotolerant *panC* group VI grew only at 20 but not at 37°C. At a temperature of 45°C, the number of growing isolates was reduced to 43 (58.7%) out of 75 and 34 of them (79.1%) were affiliated to group III. There was only one isolate of phylogenetic group III, which did not grow at 45°C. On the other side, only 9 out of 31 isolates (29.0%) belonging to *panC* group IV were able to grow at 45°C but only at a reduced rate (Figure 3). The isolates affiliated to groups II or VI were not able to grow at 45°C. In addition, no isolate could grow at 55°C. Screening for very low growth temperatures was done using agar plates in the refrigerator (5 to 7°C) for 7 days. There was no isolate showing any visible growth on agar plates thus precluding growth of *B. cereus* from packaging materials in properly stored goods in the refrigerator. When comparing the growth curves at 37 and 45°C, especially isolates affiliated to group III showed curve shapes with a distinct decrease in biomass after initial exponential phase. We aimed to compare growth at 20°C with 37°C for isolates affiliated to mesophilic groups II, III, and IV and we revealed an overall increased growth rate at 37°C with an OD_600_ slope of 0.0695 per 15 min compared to 0.0401 at 20°C. This implies a growth 1.7 times faster at 37°C within the exponential phase. The increased growth rate at 37°C could also be shown separately for these phylogenetic groups (Figure 4), with the difference between the slopes at 20 and 37°C being significantly more pronounced for groups III and IV than for group II. For the calculation, we selected those parts of the growth curves that had OD_600_ values between 0.4 and 0.65 as representative of the exponential growth phase. Despite an increased growth rate at 37°C, incubation at 20°C resulted in overall higher amounts of biomass in group II, III, and IV compared to 37°C with a median difference of OD_600_ = + 0.193 (74 isolates, Wilcoxon matched-pairs signed rank test, *p* < 0.0001). This could also be shown separately for averaged data of *panC* groups II, III, and IV, with median differences in OD_600_ of +0.2727, +0.1945, and +0.2112 (each *p* < 0.0001), respectively. However, differences between the three phylogenetic groups are not significant (Kruskal–Wallis test, *p* = 0.0643). Furthermore, growth at 45°C was not only limited to 58.7% of the isolates, but also resulted in further decreasing amount of biomass compared to growth at 20 and 37°C (Kruskal–Wallis test, *p* < 0.0001; Figure 5). When grown on blood agar, there were 71 out of 75 isolates (94.7%) showing beta-hemolysis in different forms ranging from slight hemolytic activity to wide hemolytic halos of several millimeters, a tendency to certain phylogenetic groups was not observed, however. Four isolates (BC2, BC20, BC55, and BC65) did not cause beta-hemolysis and are located in close proximity to each other in the same phylogenetic sub-branch within *panC* group IV.

### Toxin Gene Detection

**FIGURE 6:**
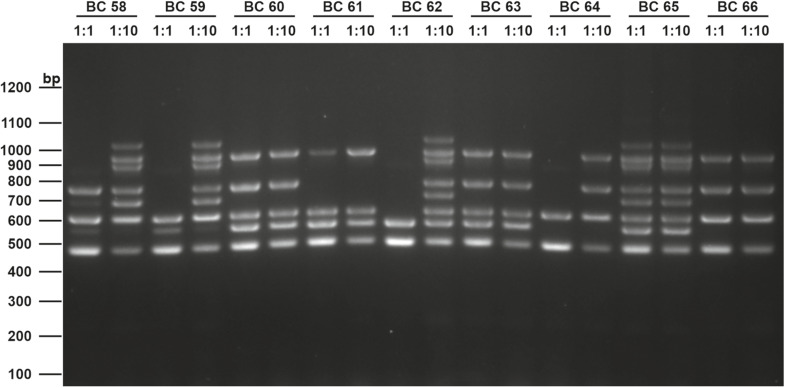
Enterotoxin multiplex PCR on 1.6% agarose gel. Following genes were included: *hblD* (1,018 bp), *hblA* (884 bp), *hblC* (695 bp), *nheB* (935 bp), *nheA* (759 bp), *nheC* (618 bp), *cytK* (565 bp), and *entFM* (486 bp). Template DNA of representative isolates used in this figure (BC58 – BC66) were tested undiluted (1:1) and diluted (1:10) to minimize PCR inhibition. A 100 bp DNA ladder was used as a marker.

The screening for enterotoxin genes (*hblDAC*, *nheABC*, *cytK*, and *entFM*) and the plasmid encoded toxin genes (*ces*, *cry1*-group genes) by a multiplex (Figure 6) and two singleplex PCR, respectively, revealed a broad heterogeneity of the isolates. While *hbl* genes were found in 38 out of 75 isolates (50.7%), the complete operon was detectable in only 33 isolates (44.0%). In five isolates bands for either *hblD*, *hblA*, or *hblC* were not detectable in the agarose gel. The *nhe* genes were found in all isolates, and in 68 out of 75 (90.7%) all three genes of the operon were detectable. Here, *nheC* but not *nheA* could be detected in some isolates and *vice-versa*. In addition, the *cytK* gene was present in 50 isolates (66.7%) and *entFM* gene was detected in all isolates. Four isolates (5.3%) carried the *ces* gene, whereas none of the isolates carried a gene for *cry1* group crystal toxins. PCR products for each target gene were sequenced once and confirmed correct PCR amplification. In total, 13 different toxin gene profiles could be established (Table 4), which partially correspond to previously published toxin gene profiles of *B. cereus* s.l. isolates (Ehling-Schulz et al., 2006; Carter et al., 2018). Toxin profile E (*hbl^–^, nhe^+^, cytK^+^, entFM^+^*, and *ces*^–^) was the most abundant with 23 isolates (30.7%), followed by profile A (*hbl^+^, nhe^+^, cytK^+^, entFM^+^*, and *ces*^–^) with 22 isolates (29.3%). Isolates possessing the *ces*-gene were negative for *hblDAC* and *cytK*. Furthermore, the emetic isolates were found in samples from manufacturers 1 and 6. Those four isolates not showing any hemolytic activity on blood agar were assigned to toxin profiles A (1) and E (3), thus questioning the actual role of individual enterotoxins in blood agar hemolysis.

**TABLE 4:** Toxin profiles of *B. cereus* group isolates.

Profile	Target gene										No. of samples
	
	*hblD*	*hblA*	*hblC*	*nheB*	*nheA*	*nheC*	*cytK*	*entFM*	*ces*	*cry1-*type	
A	(+)	(+)	(+)	(+)	(+)	(+)	(+)	(+)	(–)	(–)	22
B	(+)	(+)	(+)	(+)	(+)	(+)	(–)	(+)	(–)	(–)	7
C	(+)	(–)	(+)	(+)	(+)	(–)	(–)	(+)	(–)	(–)	1
D	(–)	(–)	(–)	(+)	(+)	(+)	(–)	(+)	(–)	(–)	8
E	(–)	(–)	(–)	(+)	(+)	(+)	(+)	(+)	(–)	(–)	23
F	(–)	(–)	(–)	(+)	(–)	(+)	(+)	(+)	(–)	(–)	2
G	(+)	(+)	(–)	(+)	(+)	(+)	(+)	(+)	(–)	(–)	1
H	(–)	(+)	(+)	(+)	(+)	(+)	(+)	(+)	(–)	(–)	1
I	(+)	(+)	(+)	(+)	(–)	(+)	(+)	(+)	(–)	(–)	1
J	(+)	(+)	(+)	(+)	(–)	(+)	(–)	(+)	(–)	(–)	3
K	(+)	(+)	(–)	(+)	(+)	(+)	(–)	(+)	(–)	(–)	1
L	(–)	(–)	(+)	(+)	(+)	(+)	(–)	(+)	(–)	(–)	1
M	(–)	(–)	(–)	(+)	(+)	(+)	(–)	(+)	(+)	(–)	4
											*n* = 75

*Thirteen different toxin profiles (A – M) were detected by multiplex PCR (enterotoxin genes) and two singleplex PCRs (*ces*, *cry1*-type genes). (+) indicates successful amplification.*

## Discussion

Although *B. cereus* has been frequently isolated from numerous sources, its occurrence in packaging materials has been poorly investigated. We reported *B. cereus* in the majority of packaging material samples included in this study. Interestingly, samples from different manufacturing sites showed different loads of *B. cereus*, but it is unclear whether these results are attributable to differences in the production site environment (geographical location, raw materials, and process technology) or to specific properties of the packaging material itself. Nevertheless, the load of *B. cereus* appears to be significantly lower in primary packaging than in secondary packaging. The mere detection of *B. cereus* in packaging material is not surprising and has already been described several times. Suihko et al. (2004) isolated *B. cereus* from packaging materials in Finland and Pirttijarvi et al. (1999) found 16 different *B. cereus* ribotypes in packaging materials from four different countries in Europe and North America. The presence of *B. cereus* in packaging materials nevertheless appears to be negligible, because countless studies showed that *B. cereus* can be isolated from raw food such as fresh vegetables (Fiedler et al., 2019) as well as ready–to-eat meals (Kotzekidou, 2013; Yu et al., 2020). Furthermore, a transfer of *B. thuringiensis* spores from food packaging to rice and chocolate was demonstrated only at very low rates (0.001 to 0.03%). We also aimed to characterize 75 different isolates from 41 packaging material samples. Although only isolates from the manufacturers with the highest abundance of *B. cereus* were investigated, our study provides the first solid overview of *B. cereus* in packaging materials and suggests that the results are transferable to other packaging manufacturers as well. Established differentiation schemes for the *B. cereus* group are either too unreliable like different colony morphologies and plasmid encoded toxin genes or often do not allow precise species identification, e.g., MALDI-TOF. Most MALDI-TOF systems, like the one used in this study, are designed for clinical diagnostics and can therefore only assign species to the *B. cereus* group without more precise identification. This emphasizes the importance of molecular methods. Sequencing of the partial *panC* gene is a fast and reliable approach to cluster members of the *B. cereus* group into seven phylogenetic groups (Guinebretière et al., 2008). We found species affiliated to four different phylogenetic groups with mesophilic groups III and IV prevailing and groups with psychrotolerant tendency (II, VI) were the minority. Studies showed a predominance of group III isolates in powdered infant formula (Heini et al., 2018) and other food products (Amor et al., 2018) as well as stated their role in food poisoning outbreaks (Glasset et al., 2016), followed by group IV. While members of groups III and IV were detected at equal frequencies in raw materials and processing environments of powdered infant formula (Zhuang et al., 2019), over half of all strains in a study on mostly environmental isolates from soil and animal feces belonged to group IV and all the food poisoning isolates to group III (Okutani et al., 2019). An epidemiologic and genetic survey revealed groups II, III, and IV in 23, 47, and 30% of patient and hospital environment strains, respectively, resembling the distribution in packaging material (Glasset et al., 2018). Therefore, the population of industrial products is likely to be between environmental and human-associated isolates, a conceivable hypothesis considering the presence of natural resources and humans in most industrial production fields. Due to the uneven distribution of the samples’ origin, manufacturing sites 6 and 1 accounted for 69.3 and 20% of all isolates, respectively, it remains ambiguous if a distinct *panC* group predominates in a packaging material facility, but the results clearly suggest that the mesophilic *panC* groups III and IV prevail in all facilities. In addition to the seven major phylogenetic groups, Guinebretière et al. (2008) proposed 14 subgroups but an exact affiliation to subgroups was not made, although some subgroups are seen as particularly dangerous for food poisoning (Guinebretière et al., 2010). Surveillance of the *B. cereus* group in the food industry has become more important, since growth in stored food is a major cause of food spoilage and psychrotolerant strains that can grow at temperatures from 5 to 10°C are of particular concern. In this study, we did not find any isolate that showed visible growth on blood agar after 7 days in the refrigerator (5 to 7°C). This, together with the low frequency of isolates belonging to psychrotolerant groups and the limited transfer of bacterial spores from paper to food (Ekman et al., 2009), strongly suggests that there is minimal risk for properly stored food. Nevertheless, more microbiological research on the interaction of packaging materials with packaged goods is needed, as literature in this field is scarce. To examine growth curves at different temperatures, we performed growth monitoring in the Bioscreen C for mesophilic and thermotolerant species and generally confirmed the growth characteristics previously associated with the phylogenetic *panC* groups (Guinebretière et al., 2008). Moreover, we showed for mesophilic isolates that growth rates in the exponential phase at 37°C are significantly increased compared to 20°C, which is, however, associated with an overall reduced maximum biomass. Higher temperatures may promote faster growth, and it has long been known that the optimal growth temperature is about 6°C below maximal growth temperature for many *Bacillus* species (Warth, 1978). Increased biomass at lower temperatures (10°C) has recently been shown for mesophilic food-associated bacteria, but only a single *B. cereus* isolate was tested (Seel et al., 2016). Our study can therefore support these results with a larger number of isolates. Limited biomass production at optimal growth temperatures may result from increased efforts to maintain cellular functions, as thermal instability of proteins appears to be a driving force in the adaption of life to different temperatures and habitats (Corkrey et al., 2012). Lower temperatures thus compensate detrimental effects in spite of challenging effects like reduced enzyme activity and low transcriptional and translational rates (D’Amico et al., 2006). The diversity within the *B. cereus* group is also reflected in their varying pathogenicity, which is mostly based on the production of several chromosomal or plasmid encoded toxins. Our results are generally in accordance with other findings of *B. cereus* group strains from various sources, where *nhe* genes were more prevalent than *hbl* genes (Guinebretière et al., 2002; Ngamwongsatit et al., 2008; Yu et al., 2020). This is supported by the phylogenetics of enterotoxins, which provided evidence, that gene deletion, duplications and horizontal gene transfer is likely for *hbl* genes, whereas *nhe* gene follow vertical inheritance (Böhm et al., 2015). Nevertheless, some studies also showed *hbl* genes prevailing in food strains, thus no universal conclusion can be drawn. The CytK prevalence varies between different studies and generally matches with our findings. The actual role of EntFM in *B. cereus* remains still unclear, as it is not sufficiently understood whether it serves as an enterotoxin or as a cell wall peptidase (Asano et al., 1997; Tran et al., 2010). Overall, we discovered 13 different toxin profiles, some of which are consistent with previously published toxin gene profiles (Ehling-Schulz et al., 2006; Carter et al., 2018; Fiedler et al., 2019). The link between *panC* group and toxin genes has already been shown and could be confirmed by this study (Guinebretière et al., 2010). Although *panC* sequencing does not precisely distinguish the species within a phylogenetic group, combining the results with those from toxin PCRs can lead to species definitions that are more accurate. According to the literature, *B. thuringiensis* is defined by the presence of the plasmid encoded crystal toxins (Schnepf et al., 1998). Since we could not detect any isolate carrying this plasmid, we concluded that all isolates within groups II, III, and IV are likely *B. cereus sensu stricto* and not *B. thuringiensis*. In addition, the isolate affiliated to group VI is probably *B. weihenstephanensis* because it did not carry the crystal toxin plasmid and did not show rhizoid colony morphology typical for *B. mycoides*. Emetic strains were detected only at low rates in packaging materials and have been reported at comparable rates in food samples by other studies (Ehling-Schulz et al., 2006; Heini et al., 2018; Yu et al., 2020). The fact that emetic strains were isolated in samples from two manufacturers showed that they do not occur exclusively in one packaging type, but no link to manufacturing conditions or sample properties can be made due to lack of data. In accordance with other enterotoxin screenings, not all genes of the *hblDAC* and the *nheABC* operons were detectable for each isolate. While several other studies also indicated these strains to have individual toxin profiles, it is unlikely that this is caused by the absence of these genes, but rather by sequence polymorphisms that prevent accurate primer annealing (Guinebretière et al., 2002). Consequently, PCR strategies that involve the detection of single genes have greater potential for false-negative results compared to multiplex assays that attempt to amplify each gene. Notwithstanding the phylogenetic affiliation is a simple approach to characterize isolates, toxin detection remains irreplaceable due to more and more reports of formerly less virulent *B. cereus* group species harboring enterotoxin genes (Prüß et al., 1999; Baron et al., 2007) as well as cereulide (Thorsen et al., 2006). Moreover, it has been suggested, that the *B. cereus* group only comprises lineages of the same species possessing different growth characteristics and virulence factors such as toxins encoded on plasmids (Helgason et al., 2000; Rasko et al., 2005). This indicates that several characteristics need to be taken into consideration to evaluate *B. cereus* group strains. This study shows that packaging material isolates share the same characteristics with strains from food or environmental sources and therefore packaging materials may not be a reservoir for particularly virulent strains.

## Data Availability Statement

The original contributions presented in the study are included in the article/supplementary material, further inquiries can be directed to the corresponding author/s.

## Author Contributions

PS: experimental design, data collection, analysis, and interpretation, and manuscript writing. SM: data collection and manuscript writing. CK: project supervision and manuscript writing. All authors have approved the final version to be published and agreed to be accountable for all aspects of the work in ensuring that questions related to the accuracy or integrity of any part of the work are appropriately investigated and resolved.

## Conflict of Interest

The authors declare that the research was conducted in the absence of any commercial or financial relationships that could be construed as a potential conflict of interest.

## Footnotes

1 https://www.tools.symprevius.org/Bcereus/
